# Electrophysiological Correlates of Processing Warning Signs With Different Background Colors: An Event-Related Potentials Investigation

**DOI:** 10.3389/fpsyg.2021.648871

**Published:** 2021-04-20

**Authors:** Jingpeng Yuan, Zhipeng Song, Ying Hu, Huijian Fu, Xiao Liu, Jun Bian

**Affiliations:** ^1^Zhejiang Zheshang Engineering Service of Equipment Co., LTD., Hangzhou, China; ^2^Zhejiang Provincial Economic Construction Investment Co., LTD., Hangzhou, China; ^3^Department of Traffic Information, Zhejiang Expressway Information Engineering and Technology Co., LTD., Hangzhou, China; ^4^ZEIET Research Institute, Hangzhou, China; ^5^Neuromanagement Lab, Zhejiang University, Hangzhou, China; ^6^Zhejiang State-owned Capital Operation Co., LTD., Hangzhou, China; ^7^School of Management, Guangdong University of Technology, Guangzhou, China

**Keywords:** warning signs, background color, hazard perception, event-related potentials, safety signs

## Abstract

Warning signs, as a type of safety signs, are widely applied in our daily lives to informing people about potential hazards and prompting safe behavior. Although previous studies have paid attention to the color of warning signs, they are mostly based on surveys and behavioral experiments. The neural substrates underlying the perception of warning signs with different background colors remain not clearly characterized. Therefore, this research is intended to address this gap with event-related potentials (ERPs) technique. Warning signs with three different background colors (i.e., white, yellow, and blue) were used in the experiment. The results showed that the perceptual differences between different warning signs were present in the form of differential ERPs components (P1, N1, P2, N2, and P3) though subjects were not required to explicitly attend to the warning signs.

## Introduction

Colors are widely used in our daily lives and play important roles other than simple decoration. They not only help people to distinguish different objects but also convey messages. For example, in general, a red traffic light means “stop,” a green traffic light means “go,” and a yellow traffic light means “take caution.” In the Chinese stock market, red color signals the rise in stock price, while green signals the fall of stock price. Yet in America, the meanings of colors in the stock market are opposite to those in China. Therefore, different colors are perceived differently, depending on the context where they are used. Given that colors are capable of conveying hazard or risk information intuitively, they are considered as a main design element of safety signs (Edworthy and Adams, 1996; ANSI, 2007; GB2894, 2008).

As a type of safety sign, warning sign is a prominent tool used to remind people of potential hazards and instruct them to behave safely. A lot of effort has been devoted to the design and effectiveness of warning signs in the past decades (Chan and Ng, 2010; Ma et al., 2010, 2018; Chan and Ng, 2012; Bian et al., 2020; Zhu et al., 2020). In this research trend, the color of warning signs has also received considerable attention because color is essential when the textual message is restricted (Chapanis, 1994; Braun et al., 1995; Borade et al., 2008; Siu et al., 2017; Chen et al., 2020). White, yellow, orange, and red colors are found to be associated with successively greater perceived hazard levels (Chapanis, 1994). Colored warning labels are regarded as more readable and more hazardous than black-and-white warning labels (Braun et al., 1995). Braun and Shaver (1999) studied warning signs with different colors behind the signal word (red vs. blue) and different levels of text explicitness (none vs. low vs. high), and noted that red warnings resulted in higher hazard ratings than blue warnings only when the text explicitness was high. Luximon et al. (2003) suggested that warning signs with red background, black border, and white text led to the highest danger rating, while white background, blue border, and black text led to the highest information rating. A recent study revealed that red warning signs were easier to be identified and understood than yellow and black signs (Chen et al., 2018). Based on the Indian population, Borade et al. (2008) demonstrated that certain colors signaled different hazard levels and suggested that population factors be considered in hazard communication. Moreover, it is indicated that a fire safety evacuation sign with “green and black” color leads to the lowest cognitive load, highest search efficiency, and best evacuation escape performance (Chen et al., 2020).

Drawing from extant literature on sign colors, it could be seen that the findings are mixed and more research is needed to figure out which color is most effective for warning signs to communicate hazard information. Meanwhile, since prior studies on sign colors mainly adopted interviews, surveys, and behavioral experiments (Wogalter and Laughery, 1996; Rogers et al., 2000; Laughery, 2006; Williams and Noyes, 2007), relatively little is known about the neural correlates of how people perceive warning signs with different colors. Therefore, this research took a preliminary step and focused on exploring the neural processes underlying the perception and cognition of warning signs with different background colors with event-related potentials (ERPs) technology.

Event-related potentials provide direct measures of perceptual and cognitive processes with high temporal resolution. Amplitudes of ERPs components are supposed to represent the degree of engagement of cognitive processes, and latencies of them indicate the time stages of information processing (Luck, 2005). Among ERPs components, P1, N1, and P2 are considered to index relatively early perceptual stages of information processing, while N2 and P3 (including P300 and LPP) are considered to index relatively late, elaborate and high-level cognitive process, with P and N indicating whether a component is positive-going or negative-going and the number indicating a component’s ordinal position within the waveform (Thomas et al., 2007; Lu et al., 2010). P1 typically arises at about 100 ms after stimulus onset and is associated with the physical features of the stimulus (Vogel and Luck, 2000; Zhao, 2010; Kendall et al., 2016). A Stimulus with high contrast and reduced complexity evokes smaller P1 amplitude than a stimulus with low contrast and increased complexity (Hosseinmenni et al., 2015; Kendall et al., 2016). Also sensitive to the visual features, N1 indicates the discrimination and classification of stimuli (Eimer, 2000; Vogel and Luck, 2000). For instance, the discrimination between facial and non-facial stimuli and between stimuli with different emotional valence is found to induce pronounced N1 component (Eimer, 2000; Frühholz et al., 2011). Frontal P2 is related to attentional processing and working memory manipulations (Holmes et al., 2008; Lu et al., 2010). A larger P2 amplitude is found to be evoked by objects with appropriate color than those with inappropriate color, suggesting that the perceptual memory about natural color is activated during the relatively early stage of information processing (Lu et al., 2010). Frontal N2 has been reported to be indicative of stimulus classification (Cao et al., 2010; Lu et al., 2010; Nittono et al., 2010). Moreover, P3 reflects attention allocation related to stimulus evaluation and categorical processing (Polich, 2007; Holmes et al., 2008). Categorization of stimuli along evaluative or non-evaluative dimensions has been revealed to be associated with P3 component (Cao et al., 2010; Fu et al., 2017).

Prior research has shown that ERPs are conducive to understanding how people process safety signs (Fu et al., 2017; Ma et al., 2018; Lu and Hou, 2019; Bian et al., 2020; Zhu et al., 2020). For instance, warning signs with higher hazard levels are associated with increased N1, N2, and P300/LPP components (Ma et al., 2010, 2018; Fu et al., 2017; Bian et al., 2020), and a reduced P2 component (Ma et al., 2018; Bian et al., 2020). In addition, ERPs have also been employed to study the cognitive processing of different colors (Holmes et al., 2008; Cano et al., 2009; Cao et al., 2010; Lu et al., 2010). For example, Cao et al. (2010) explored how people process yellow and blue colors and noticed that larger N1, P2, N2, and P3 components were evoked by yellow (vs. blue) stimulus. Cano et al. (2009) reported that affective valence had an effect on P3 component when the image was in color, but did not when the image was in black-and-white. Color knowledge affects early object recognition stages, such that N1, P2, and N2 components differ between objects in their appropriate colors and objects in inappropriate colors (Lu et al., 2010).

In this study, we attempts to extend previous studies on warning signs by investigating how people perceive warning signs with different background colors with an implicit paradigm. Recent research suggests that an implicit paradigm that does not require participants to explicitly pay attention to the safety signs is feasible to study how people perceive these signs, since they might be processed implicitly in many real life cases (Ma et al., 2018; Bian et al., 2020). Such a paradigm is not only helpful in examining the automatic information processing driven by stimuli, but also in avoiding a “relevance-for-task” effect (Bian et al., 2020; Zhu et al., 2020). Accordingly, the electroencephalogram (EEG) experiment in the present study adopted an implicit paradigm. A questionnaire-based experiment was performed in advance to collect subjective data, which provided an important complement to the EEG data. Neurophysiologically, we expect that warning signs with different background colors will lead to differential perceptual (P1, N1, and P2) and cognitive (N2 and P3) ERPs.

## Materials and Methods

### Subjects

Eighteen subjects (six females) aged between 19 and 34 years (M ± SD = 23.06 ± 4.29) were recruited from Zhejiang University as paid volunteers. All subjects were healthy, right-handed native speakers with normal or corrected-to-normal vision. Meanwhile, they reported to be free of any history of neurological disorders and mental diseases. The protocol of this study complied with Declaration of Helsinki and was approved by the Internal Review Board of the Neuromanagement Lab in Zhejiang University. Each participant provided a written informed consent before the formal experiment started. Data from one male subject was discarded due to excessive recording artifacts, leading to 17 valid subjects (six females) for final analysis.

### Materials

**Figure 1:**
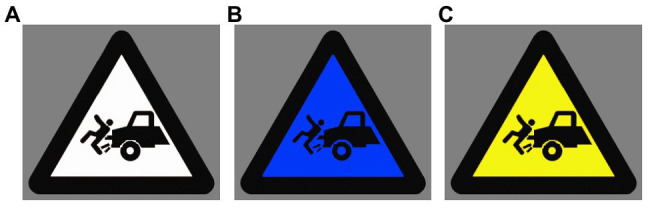
Examples of experimental stimuli. A warning sign with **(A)** white, **(B)** blue, and **(C)** yellow background.

This study attempted to examine the implicit processing of warning signs with three different background colors (i.e., white, blue, and yellow). Twelve pictures of warning signs with yellow background were selected according to the Chinese National Standard for safety signs (GB2894, 2008). Adobe Photoshop CS3 image processing software (Adobe Systems Incorporated, San Jose, California, United States) was used to alter the background color of these signs. Hence three groups of warning signs were obtained with different background colors but identical pictorials and surrounding shapes (see Figure 1 as an example). Accordingly, three main conditions were created, i.e., warning signs with white, blue and yellow backgrounds (hereafter also referred to as white, blue and yellow signs). The quality, size and resolution of pictures remained consistent across conditions. A questionnaire-based experiment was conducted to collect self-report data on people’s perception of these signs. One hundred and twenty-four respondents who did not participate in the EEG experiment were randomly assigned to one of the three conditions. They were asked to rate the perceived hazard level and readability of each sign on seven-point Likert scales (1 = very low, 7 = very high; Braun et al., 1995). The respondents were also required to indicate the background color of warning signs in the Chinese national standard and to indicate which background color was most feasible for warning signs in their opinion.

In the EEG experiment, however, warning signs were used as non-target stimuli and subjects were not asked to attend to the signs. Twelve neutral pictures of chairs were selected as target stimuli and subjects were required to count the number of chairs presented in each block. During the experiment, each picture was repeated three times. Therefore, there were 144 trials in total, with 36 trials in each condition (i.e., white signs, blue signs, yellow signs, and chairs).

### Procedure

The experimental procedure was similar to the study conducted by Bian et al. (2020). Subjects were seated comfortably in a dimly lit, sound-attenuated and electrically shielded room with the computer screen positioned approximately 100 cm in front of them. The experimental procedure was introduced on paper handouts prior to the formal experiment. Subjects were informed that they would be shown a number of stimuli in each block and the experiment was intended to assess their accuracy in memorizing the number of target stimuli. They were also told that the payments for their participation were linked to their performances in the experiment. Each subject had a practice session with 10 trials to get familiar with the experimental procedure.

**Figure 2:**
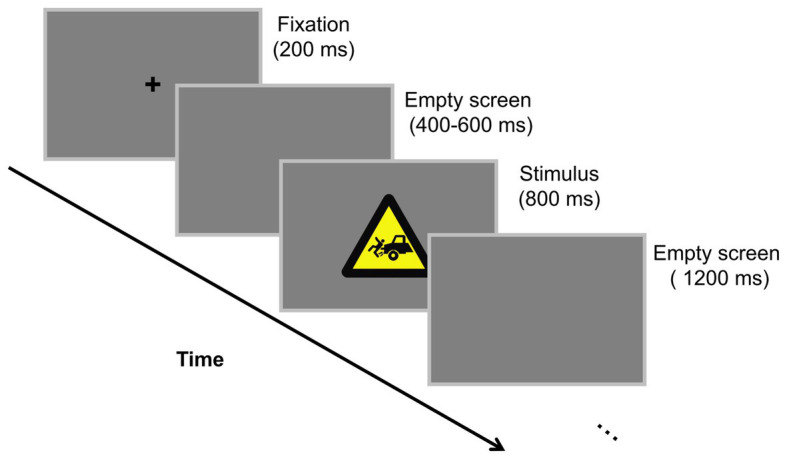
Experimental scheme. Subjects were asked to count the number of chairs presented in each block in their mind.

Subjects had to complete three blocks in the formal experiment, with 48 pseudorandomized trials in each block. The stimuli were presented at the center of a gray screen. As Figure 2 displayed, each trial began with a cross presented for 200 ms, following that was an interval with a random duration between 400 and 600 ms. Then, a target or non-target stimulus was presented for 800 ms, which was followed by an inter-trial interval lasting for 1,200 ms. Subjects were required to count the number of target stimulus (chairs) in each block in their minds and to report the number upon completion of the block. By this mean, subjects were prompted to focus their attention on the target stimuli, rendering the processing of warning signs task-irrelevant (Ma et al., 2018; Bian et al., 2020; Zhu et al., 2020). Data from a subject would be excluded from final analysis if he (or she) got the numbers wrong in more than one block. In fact, no subjects made mistakes in two or more blocks. Hence, the performance of all subjects was deemed to be acceptable and each of them was paid for 30 RMB as a financial reward at the end of the experiment.

### Electroencephalogram Data Acquisition and Analysis

Scalp EEG was recorded continuously with an electrode cap with 64 Ag/AgCl electrodes mounted according to the extended international 10–20 system. Data were sampled at 1,000 Hz using Neuroscan Synamp2 Amplifer (Scan 4.3.1, Neurosoft Labs, Inc. Sterling, United States), with online band-pass-filtered from 0.05 to 100 Hz. Electrooculogram (EOG) was recorded from electrodes placed 1.0 cm lateral to the external canthi of both eyes (horizontal EOG), and above and below the left eye (vertical EOG). A cephalic location was applied as the ground and the left mastoid served as on-line reference. Impedances were kept below 5 KΩ throughout the experiment.

During off-line processing, channel data were re-referenced to the average of the left and right mastoids and corrected for excessive eye movement using the Semlitsch et al. (1986) procedure. Stimulus-locked EEG data were digitally filtered with a bandpass from 0.1 to 30 Hz (24 dB/Octave) and segmented into epochs comprised of 200 ms before stimulus onset and 800 ms after the onset. Afterward, data were baseline-corrected by the 200 ms pre-stimulus interval. Trials contaminated by amplifier clipping, bursts of electromyographic activity, or peak-to-peak deflection exceeding ±80 μV were excluded from averaging. Only non-target stimuli (warning signs) were analyzed in this study. Therefore, EEGs over each recording site were averaged for white, blue, and yellow signs separately for each subject. These data were then grand averaged for each condition.

Based on visual inspection of the grand averaged waveforms and prior research on safety sign and color perception, five components of ERPs, P1, N1, P2, N2, and P3, were quantified from the individual participants’ waveforms. Eight electrodes (P7, P8, PO7, PO8, CB1, CB2, O1, and O2) in the parietal and occipital regions were selected for P1 and N1 analyses. Six electrodes (F3, Fz, F4, FC3, FCz, and FC4) in the frontal and fronto-central regions were selected for P2, N2, and P300 analyses. The mean amplitudes in the time windows of 100–120 and 150–170 ms were computed for P1 and N1, respectively, before being submitted to 3 (color: white, blue, and yellow) × 8 (electrode: P7, P8, PO7, PO8, CB1, CB2, O1, and O2) repeated measure ANOVAs. Similarly, the mean amplitudes in the time windows of 145–165, 215–245, and 350–380 ms were calculated for P2, N2, and P300, respectively, before entering 3 (color: white, blue, and yellow) × 6 (electrode: F3, Fz, F4, FC3, FCz, and FC4) repeated measure ANOVAs. The Greenhouse–Geisser correction was applied in case of violation of the sphericity assumption and the Bonferroni correction was used for multiple paired comparisons.

## Results

### Self-Repot Results

Among the 124 respondents who took part in the questionnaire-based experiment, 57.3% of them were female and 42.7% were male, with age ranges of 18–25 (25.8%), 26–30 (24.2%), 31–40 (33.1%), 41–50 (12.1%), and larger than 50 (4.8%). The ANOVAs on perceived hazard level [*F*(2,121) = 4.523, *p* = 0.013, *η*^2^*_p_* = 0.070] and readability [*F*(2,121) = 4.731, *p* = 0.011, *η*^2^*_p_* = 0.073] showed significant main effects of color. As illustrated in Table 1, pairwise comparisons showed that yellow and white signs were perceived to be associated with higher level of hazard and to be more readable than blue signs. Seventy-nine percent of the respondents correctly indicate that yellow was the background color for warning signs in the Chinese national standard, while 11.3% of them thought that white was the one and 9.7% of them thought blue was the one. Moreover, the percentage of the respondents that indicated yellow as the most feasible background color for warning signs was 79.8%, while the percentages for white and blue were 11.35 and 8.9%, respectively.

**Table 1:** Summary of self-report results.

	white		blue		yellow		Pairwise comparison results
	M	S.E.	M	S.E.	M	S.E.	
hazard level	5.815	0.122	5.442	0.119	5.935	0.124	yellow > blue^*^, white > blue^’^
readability	5.634	0.157	5.112	0.153	5.665	0.147	yellow > blue^*^, white > blue^*^

’ *p* < 0.1;

* *p* < 0.05.

### ERPs Results

#### P1 Analysis

**Figure 3:**
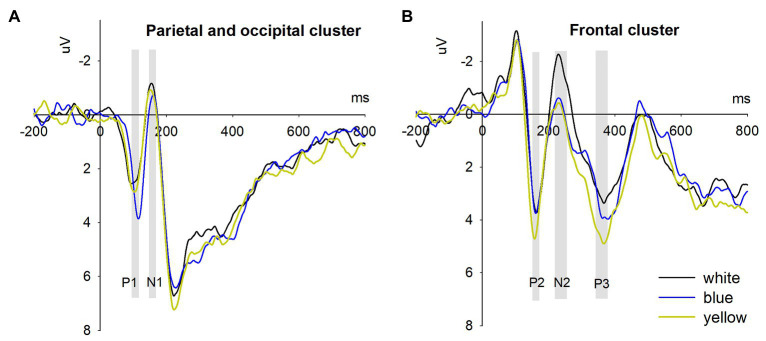
Event-related potentials results. **(A)** The parietal-and-occipital cluster represents the averaged waveforms across eight electrodes (P7, P8, PO7, PO8, CB1, CB2, O1, and O2); **(B)** The frontal cluster represents the averaged waveforms across six electrodes (F3, Fz, F4, FC3, FCz, and FC4).

The ERPs grand averaged waveforms at two representative clusters (parietal-and-occipital cluster and frontal cluster) are displayed in Figure 3. The ANOVA on P1 amplitude showed that the main effect of color [*F*(2,32) = 5.874, *p* = 0.014, *η*^2^_*p*_ = 0.269] was significant. As illustrated in Table 2, the amplitude of P1 component induced by blue signs (*M* = 3.493 μV, S.E. = 0.472) was larger than that induced by yellow signs (*M* = 2.596 μV, S.E. = 0.416, *p* = 0.033) and white signs (*M* = 2.311 μV, S.E. = 0.448, *p* = 0.057). But there was no significant difference in P1 amplitude between the yellow and white signs (*p* = 1.000). The main effect of electrode was significant [*F*(7,112) = 3.859, *p* = 0.033, *η*^2^*_p_* = 0.194], but the interaction between color and electrode was not [*F*(14,224) = 1.451, *p* = 0.225, *η*^2^*_p_* = 0.083].

**Table 2:** Summary of event-related potentials (ERPs) results.

	white		blue		yellow		Pairwise comparison results
	M (μV)	S.E.	M (μV)	S.E.	M (μV)	S.E.	
P1	2.311	0.448	3.493	0.472	2.596	0.416	yellow < blue^*^, white < blue^’^
N1	−0.814	0.778	0.132	0.805	−0.734	0.804	yellow > blue^’^, white > blue^*^
P2	3.295	0.771	3.400	0.806	4.595	0.947	yellow > blue^’^, yellow > white^*^
N2	−2.132	0.972	−0.543	0.790	−0.342	0.858	white > blue^*^, white > yellow^*^
P3	3.258	1.356	3.887	1.423	4.798	1.237	yellow > white^**^

P1, P2, and P3 refer to the first, second, and third positive components after stimulus onset, N1 and N2 refer to the first and second negative components after stimulus onset.

’ *p* < 0.1;

* *p* < 0.05;

** *p* < 0.01. The relative magnitudes of an ERPs component in different conditions were determined depending on its polarity.

#### N1 Analysis

The ANOVA on N1 amplitude indicated a significant main effect of color [*F*(2,32) = 5.566 μV, *p* = 0.008, *η*^2^*_p_* = 0.258]. Blue signs (*M* = 0.132 μV, S.E. = 0.805) led to a smaller N1 than yellow signs (M = −0.734 μV, S.E. = 0.804, *p* = 0.065) and white signs (*M* = −0.814 μV, S.E. = 0.778, *p* = 0.034). But there was no significant difference between yellow and white signs (*p* = 1.000). Moreover, the main effect of electrode [*F*(7,112) = 0.600, *p* = 0.627, *η*^2^*_p_* = 0.036] and the interaction between color and electrode [*F*(14,224) = 1.672, *p* = 0.196, *η*^2^*_p_* = 0.095] were not significant.

#### P2 Analysis

The results showed that the main effect of color [*F*(2,32) = 5.162, *p* = 0.01, *η*^2^*_p_* = 0.244] was significant. Yellow signs (*M* = 4.595 μV, S.E. = 0.947) evoked a larger P2 than blue signs (*M* = 3.400 μV, S.E. = 0.806, *p* = 0.071) and white signs (*M* = 3.295 μV, S.E. = 0.771, *p* = 0.020). But there was no significant difference between the blue and white signs (*p* = 1.000). The main effect of electrode [*F*(5,80) = 2.340, *p* = 0.094, *η*^2^*_p_* = 0.128] and the interaction between color and electrode [*F*(10,160) = 0.813, *p* = 0.517, *η*^2^*_p_* = 0.0458] were not significant.

#### N2 Analysis

The ANOVA on N2 amplitude revealed a significant main effect of color [*F*(2,32) = 5.447 μV, *p* = 0.009, *η*^2^*_p_* = 0.254]. White signs (*M* = −2.132 μV, S.E. = 0.972) elicited a more negative N2 amplitude than yellow signs (*M* = −0.342 μV, S.E. = 0.858, *p* = 0.050) and blue signs (*M* = −0.543 μV, S.E. = 0.790, *p* = 0.048). But N2 did not differentiate between yellow and blue signs (*p* = 1.000). The main effect of electrode [*F*(5,80) = 9.733, *p* = 0.000, *η*^2^*_p_* = 0.378] and the interaction between color and electrode [*F*(10,160) = 2.211, *p* = 0.020, *η*^2^*_p_* = 0.121] were significant. Follow-up simple contrasts indicated that the simple main effects of color were significant for electrodes in the left (F3 and FC3, *ps* < 0.05) and middle regions (FZ and FCZ, *ps* < 0.05), but not for those in the right region (F4 and FC4, *ps* > 0.1).

#### P3 Analysis

The results showed that the main effect of color was significant [*F*(2,32) = 3.498, *p* = 0.042, *η*^2^*_p_* = 0.179]. Planned contrast indicated that yellow signs (*M* = 4.798 μV, S.E. = 1.237) induced a larger P3 than white signs (*M* = 3.258 μV, S.E. = 1.356, *p* = 0.007). However, P3 did not differ between yellow and blue signs (*M* = 3.887 μV, S.E. = 1.423, *p* = 0.587) and between blue and white signs (*p* = 0.985). The main effect of electrode [*F*(5,80) = 7.087, *p* = 0.001, *η*^2^*_p_* = 0.307] was significant. But the interaction between color and electrode was not significant [*F*(10,160) = 0.891, *p* = 0.543, *η*^2^*_p_* = 0.053].

## Discussion

Color, as an indispensable visible feature of safety signs, captures people’s attention easily and plays an important role in conveying information about potential hazards (Edworthy and Adams, 1996). Though prior research has endeavored to understand the effect of color on hazard perception, relatively little is known about how people perceive warning signs with different background colors in the brain. Consequently, this study is intended to delve into the neural substrates of the perception and cognition of warning signs with different background colors by electrophysiological technique. Meanwhile, an implicit paradigm, which did not require explicit attention toward warning signs was adopted to avoid a “relevance-for-task” effect (Yuan et al., 2007; Ma et al., 2018; Bian et al., 2020; Zhu et al., 2020).

### Are Perceptual and Cognitive ERPs Sensitive to Sign Color?

The ERPs results showed that both perceptual components (P1, N1, and P2) and cognitive components (N2 and P3) were evoked by warning signs with different background colors. P1 and N1 components were mainly observed in the parietal and occipital scalp regions, while P2, N2, and P3 were observed in the frontal and fronto-central regions. In general, perceptual components are indicative of earlier, lower-level and more automatic and exogenous stages of information processing and cognitive components more controlled, elaborate, and conscious cerebral activities (Thomas et al., 2007; Lu et al., 2010).

As an early attention-related perceptual component, P1 typically appears at about 100 ms after stimulus onset and is closely related to the visual features of the stimulus (Zhao, 2010; Hosseinmenni et al., 2015). P1 is sensitive to the clear and ambiguous features contained by a stimulus. For example, a smaller P1 amplitude is found to be elicited by a stimulus with high contrast and low complexity than a stimulus with low contrast and high complexity (Hosseinmenni et al., 2015; Kendall et al., 2016). In this study, compared to warning signs with blue background, those with yellow and white backgrounds resulted in decreased P1 amplitudes. This finding may be due to the fact that warning signs with different background colors differ in low-level features (e.g., visual contrast). Given their visual features, warning signs with yellow and white backgrounds are easier to be recognized than signs with blue background. The self-report results also support this notion, as respondents indicated that warning signs with yellow and white backgrounds were more readable than those with blue background.

Similar to P1 component, N1 is liable to be affected by physical features of stimuli (Hillyard and Anllo-Vento, 1998; Luck, 2005; Niu et al., 2008). N1 in the parietal and occipital scalp regions reflects discrimination and classification of stimuli. For example, as proposed by Vogel and Luck (2000), N1 induced by visual stimuli indicates the classification of stimuli at the early stage of attentional processing. Additionally, a number of studies have linked N1 to the discrimination between facial and non-facial stimuli (Eimer, 2000) and the detection of the emotional valence of the stimuli (Frühholz et al., 2011). A more negative N1 amplitude is deemed to be associated with more attention devoted to the stimuli. In the present study, warning signs with yellow and white backgrounds led to larger N1 component than those with blue background, indicating that yellow and white signs attracted more attention from subjects and were more likely to be distinguished from blue signs.

P2 in frontal region belongs to the early stage of perceptual processing and is related to the allocation of attention resources and working memory manipulations (Holmes et al., 2008; Lu et al., 2010). The natural color of objects (such as red for strawberries) is stored in the human brain as a kind of knowledge, which originates from continuous learning in people’s daily lives (Chao and Martin, 1999). Studies have shown that this color knowledge can be described and generalized as a semantic conceptual model (Lu et al., 2010). Zeki and Marini (1998) found that different pathways were adopted for processing objects dressed in natural and unnatural colors and proposed a cognitive model based on color knowledge. Based upon an implicit experimental paradigm, Lu et al. (2010) revealed that color was a part of perceptual memory, which was activated automatically during information processing. They also found notable P2 activities in the early stages of processing objects with different colors. The amplitudes of P2 are larger for objects with appropriate color than for objects with inappropriate color and with gray color (Lu et al., 2010). In this study, larger P2 was elicited by warning signs with yellow backgrounds than those with blue and white backgrounds. It might be due to that after the earlier stages of perceptual processing (P1 and N1), the semantic concepts about different colors are retrieved and warning signs with yellow background is thought to be more natural than those with blue and white backgrounds. This finding was also evidenced by the self-report data, which showed that 79.0% of the respondents who took part in the questionnaire-based experiment could correctly indicate yellow as the background color for warning signs in the Chinese national standard.

Frontal N2 component has been found to play a role in stimulus classification (Cao et al., 2010; Lu et al., 2010; Nittono et al., 2010). For instance, Cao et al. (2010) examined the neural responses toward blue and yellow objects and found a notable N2 differentiation between these two colors. Moreover, in a study comparing how people respond to objects in different colors, it is noted that objects in their appropriate color induced a smaller N2 component than objects in their inappropriate color and objects in gray color (Lu et al., 2010). In the current study, N2 amplitudes were more positive for warning signs with yellow and blue backgrounds than those with white background. Yet there was no significant difference between warning signs between blue and yellow backgrounds. We surmise that warning signs with colored background might be perceived differently from warning signs with white background at this processing stage. Though the self-report data indicated warning signs with yellow and white background was perceived as more hazardous than those with blue background, warning signs with white background might be deemed to be less appropriate for hazard communication (Braun et al., 1995).

P3 component generally indicates the allocation of attention resources related to post-perceptual stimulus evaluation and categorical processing (Polich, 2007; Holmes et al., 2008; Fu et al., 2017). Frontal P3 (or so-called P3a) has also been linked to the top–down switching of attention by frontal brain systems toward rare or physically alerting stimuli (Mccarthy et al., 1997). In a study comparing yellow and blue colors, it was found that yellow objects induced significantly larger P3 amplitude than blue objects (Cao et al., 2010). In addition, between-category stimuli could result in enhanced P3 activity compared to within-category stimuli (Holmes et al., 2008). The present study showed that an increased P3 was induced by warning signs with yellow background relative to those with white backgrounds. But no difference was found between signs with blue and white backgrounds. In consonance with extant literature, we speculate that the differentiation in P3 amplitude might be caused by categorical processing of the colors and warning signs with yellow background are thought to be more feasible for hazard communication than those with blue and white backgrounds. This interpretation was also supported by the self-report data, since a majority of respondents suggested that yellow was the most feasible background color for warning signs.

### Theoretical Significance and Practical Implication

Theoretically, this research contributes to the literature on safety sign colors from the perspective of neural processing. Prior research has mainly adopted self-reports, which is susceptible to subjective bias. This study incorporates neuroscience technology and employs an implicit paradigm, which is conducive to understanding how people process sign color without explicit attention. Moreover, by focusing on background color, this research extends the literature on sign colors. This research also has practical implications. First, warning signs with yellow background is recommended to be used instead of warning signs with white or blue backgrounds, since the former is more prone to capture people’s attention and alert people of potential hazards. Second, warning signs should be put in place where they are necessary to increase the likelihood of being processed by the audience because the present study suggests that people are able to perceive the hazards communicated by warning signs even if they do not explicitly pay attention to the signs.

### Limitations and Future Research Directions

This study is subject to several limitations, some of which may open up opportunities for future research. First, only warning signs were considered in this study to eliminate possible confounding factors resulted from including different types of safety signs. According to the message communicated by safety signs and their functions, they could be broadly classified into prohibition, mandatory, warning, and guide categories (GB2894, 2008). The Chinese national standard recommends different colors for different types of safety signs. Therefore, future studies may explore how people process sign color by using different types of safety signs. Second, this research mainly examined three background colors (i.e., white, blue, and yellow). Further research could extend this line of research and figure out people’s perception of warning signs with other background colors. Third, to offer a deeper insight into how people perceive sign colors, the colors should be tested by three primary colors of red, green, and blue as well as their combinations, and by taking the changes in brightness, hue, and saturation into consideration. The Chinese national standard fails to consider these factors, which suggests a promising avenue for future research. Fourth, the color of signal words, pictorials, and surrounding shapes are worthy of further research. Finally, the subjects of the EEG experiment were mostly undergraduate students. Their differences in safety education were not considered in the study. Further studies are warranted to recruit subjects with more diverse backgrounds to generalize the findings of the present study and to examine if safety education influences the way people perceive sign colors.

## Conclusion

Overall, this study investigates the neural correlates of how people perceive warning signs with different background colors (i.e., white, blue, and yellow) with an implicit paradigm. The results show that both perceptual components (P1, N1, and P2) and post-perceptual components (N2 and P3) are induced by signs with different background colors. These results possibly suggest that people are able to identify the differences in sign colors and the hazard information conveyed by different colors, even though their attention are not readily directed toward the signs. It may also enlighten future research on related topics.

## Data Availability Statement

The raw data supporting the conclusions of this article will be made available by the authors, without undue reservation.

## Ethics Statement

The studies involving human participants were reviewed and approved by Internal Review Board of the Neuromanagement Lab in Zhejiang University. The patients/participants provided their written informed consent to participate in this study. Written informed consent was obtained from the individual(s) for the publication of any potentially identifiable images or data included in this article.

## Author Contributions

JY and JB conceived and designed the study. JY, HF, and JB performed the experiment and analyzed the data. JY, ZS, HF, and JB interpreted the data and drafted the manuscript. JY, ZS, YH, HF, XL, and JB reviewed and refined the manuscript. JB administered the project. All authors contributed to the article and approved the submitted version.

### Conflict of Interest

JY and XL were employed by company Zhejiang Zheshang Engineering Service of Equipment Co., LTD. JY was employed by company Zhejiang Provincial Economic Construction Investment Co., LTD. ZS and JB were employed by company Zhejiang Expressway Information Engineering and Technology Co., LTD. YH was employed by company Zhejiang State-owned Capital Operation Co., LTD.

The remaining author declares that the research was conducted in the absence of any commercial or financial relationships that could be construed as a potential conflict of interest.
